# A Simple Method for Combining Genetic Mapping Data from Multiple Crosses and Experimental Designs

**DOI:** 10.1371/journal.pone.0001036

**Published:** 2007-10-17

**Authors:** Jeremy L. Peirce, Karl W. Broman, Lu Lu, Robert W. Williams

**Affiliations:** 1 Center for Neuroscience, Department of Anatomy and Neurobiology, University of Tennessee Health Science Center, Memphis, Tennessee, United States of America; 2 Center for Genomics and Bioinformatics, Department of Anatomy and Neurobiology, University of Tennessee Health Science Center, Memphis, Tennessee, United States of America; 3 Department of Biostatistics, Johns Hopkins University, Baltimore, Maryland; Duke University, United States of America

## Abstract

**Background:**

Over the past decade many linkage studies have defined chromosomal intervals containing polymorphisms that modulate a variety of traits. Many phenotypes are now associated with enough mapping data that meta-analysis could help refine locations of known QTLs and detect many novel QTLs.

**Methodology/Principal Findings:**

We describe a simple approach to combining QTL mapping results for multiple studies and demonstrate its utility using two hippocampus weight loci. Using data taken from two populations, a recombinant inbred strain set and an advanced intercross population we demonstrate considerable improvements in significance and resolution for both loci. 1-LOD support intervals were improved 51% for *Hipp1a* and 37% for *Hipp9a*. We first generate locus-wise permuted P-values for association with the phenotype from multiple maps, which can be done using a permutation method appropriate to each population. These results are then assigned to defined physical positions by interpolation between markers with known physical and genetic positions. We then use Fisher's combination test to combine position-by-position probabilities among experiments. Finally, we calculate genome-wide combined P-values by generating locus-specific P-values for each permuted map for each experiment. These permuted maps are then sampled with replacement and combined. The distribution of best locus-specific P-values for each combined map is the null distribution of genome-wide adjusted P-values.

**Conclusions/Significance:**

Our approach is applicable to a wide variety of segregating and non-segregating mapping populations, facilitates rapid refinement of physical QTL position, is complementary to other QTL fine mapping methods, and provides an appropriate genome-wide criterion of significance for combined mapping results.

## Introduction

The majority of traits that show variation based on genetic influences are affected by multiple genes of small effect as well as by environment. When compelling candidates for direct investigation are not available, QTL mapping provides an effective approach to localizing regions of the genome that are likely to mediate genetic variation in the phenotype. Mapping a QTL to an interval of 20–60 Mb has become a relatively routine matter for traits with at least moderate heritability, but narrowing the interval to a small region that includes only a few candidate genes (1–5 Mb at most) is still a highly challenging task.

A variety of methods for accomplishing the task of narrowing a QTL interval have been proposed [1]–[5] and each has advantages and disadvantages. Our complementary approach combines the results for a variety of mapping studies, each of which should add a degree of precision and significance to the final result. In this paper we describe our method and provide an implementation, which handles the problem of combining data using different markers as well as converting from genetic to physical maps and regularizing positions to be combined.

Our method calculates locus-specific P-values using the distribution of alleles at each locus. For large populations of genetically independent animals, the negative log of these locus-specific P-values is very similar to the more typically reported LOD scores, but for smaller populations like recombinant inbred strains, and non-independent populations such as advanced intercross lines, and especially for populations with missing data, they are an important innovation. For these populations, converting to locus-specific P-values is more informative and allows us to apply Fisher's combination test to combination of QTL mapping results.

Since each QTL mapping effort originates from an independently generated population, the pattern of alleles inherited in one population is independent of the pattern of alleles inherited in another population. Therefore under the null hypothesis of no linkage, locus-specific P-values for any arbitrary point on the genome are strictly independent between data sets. Given the independence of our data sets, we chose to address the problem of combining multiple testing results by using Fisher's combination test.

Since Fisher's combination test operates on P-values, our approach first defines locus-specific P-values in the original and permuted QTL maps, then combines QTL maps on a point-wise basis using Fisher's combination test. We then convert sample permuted QTL maps from each data set to locus-specific P-values, combine them, and order the best genome-wide combined P-values from these samples to define the null distribution of genome-wide adjusted P-values.

Previous efforts to meta-analyze different mapping populations have included taking the best P-value for each QTL interval and combining these values using Fisher's combination test [6] to assess the combined likelihood of ethanol drinking QTLs between C57BL/6J and DBA/2J. Unfortunately, this approach does not really increase the resolution of the QTL map, though it does improve confidence that a QTL exists in the region. Position-specific combination of QTL maps using Fisher's combination test was, however, used earlier to combine data from two different recombinant inbred (RI) strain sets [7] where the same markers were typed or could be inferred, though calculation of genome-wide adjusted significance was not considered.

Province [8] describes a correction necessary to account for truncated LOD scores in combining QTL data with the clear application of position-specific data combination by simple addition of LOD scores. Other recent investigations have used a variety of approaches to integrating genetic mapping data including standardizing common phenotypes between crosses, [9] multiple cross mapping (MCM), a method of identifying haplotype blocks that segregate as expected in an initially defined QTL interval among multiple strains, [10] and incorporation of additional data on gene density, identity [11] and expression [12].

A more recent paper by Li and colleagues [13] describes combination of multiple crosses using cross as a covariate, and outlines a permutation method that retains stratification of phenotypes by cross for determining genome-wide significance, which should work well when simple shuffling of genotypes and phenotypes within cross is sufficient to provide appropriate randomization within data set. Our approach provides a general method of integrating data even when this is not the case, for instance with Advanced Intercross Lines (AILs), [14], recombinant inbred intercrosses (RIX), and heterogeneous stocks (HS). Our approach can also be used for intercrosses (F2), backcrosses (N2), RIs and other datasets and is a simple method of integrating results from studies using individually tailored approaches like composite interval mapping. These methods can be used in a complementary manner by combining appropriate crosses using the method of Li and co-workers [13] and integrating other data sets using our method.

Our simple method also has the advantage of not requiring adjustment for use with crosses that represent different numbers of effective tests across the genome. For instance, the number of independent tests represented by an RI strain set will be greater than the number represented by a standard F2, since the genetic length of the RI genome is four times that of the F2 genome. [15] AIL and HS data sets may have even more extreme expansions of the genetic length of their genomes. Since a permuted QTL map is sampled from each permuted data set and combined in the same way as in the original combined data set, the effective number of tests in the permutations will be equivalent to that in the original data, so the genome-wide adjustment will be valid.

## Results

### Distribution of locus-specific P-values

Under the null hypothesis of no genetic effect at a locus, the distribution of P-values should be constant with a range of 0 to 1 [16]. In fact, across permutations at a given locus this is enforced in the locus-specific P-value computation by ranking of values at each locus between permutations, which results in an even distribution of values at each position. In the permutations, where **no** locus should be associated with a real genetic effect, this should be the case among loci as well, and the distribution of P-values for each permutation should also be constant and range from 0 to 1. This is the case in a variety of tested permutations (RI, F2, and AIL populations) at tested markers. Interpolation of P-values between markers does seem to slightly reduce the number of P-values observed at the very low and high ends of the distribution, however.

### The value of locus-specific P-values

**Figure 1:**
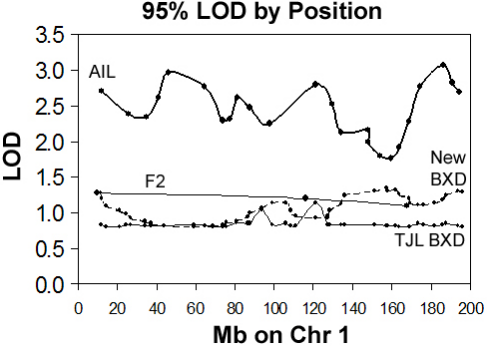
The need for locus-specific P-values. The 95% LOD score (the LOD score equivalent to a locus-specific P = 0.05) was calculated using 10,000 permutations for markers on Chr. 1 for body weight in several different populations. Each marker is indicated by a dot with connecting lines interpolated between adjacent markers. TJL BXD are BXD strains available from The Jackson Laboratory (The BXD strains developed by Taylor and colleagues [26], [27]). New BXD are the recently developed BXD strains currently resident at UTHSC. [18] Note that the maximum and minimum values of the 95^th^ percentile LOD score vary considerably for the AIL population, somewhat for the RI (New BXD and TJL BXD) populations, (predicted by missing data pattern) and very little for the 183 member F2 population tested. (There are only three widely spaced markers genotyped for the F2 population on Chr. 1, so the interpolation between points should not be interpreted as a meaningful line. However, markers on all chromosomes were very similar, between a 95% LOD of 1.2 and 1.4.)

In large data sets with independent observations—most F2 populations, for instance—the distribution of P-values under the null hypothesis of no linkage is approximately the same for each analyzed locus in the genome. The equivalent distribution of P-values by locus under the null hypothesis means that any two positions in the genome are equally likely to be the best P-value in the genome in a given permutation of an original data set. This assumption does not always hold in small data sets such as RI populations and populations with non-independent observations such as AILs. (Fig. 1) For consistency, however, we calculate locus-specific P-values for all data considered in this paper.

### Locus-specific P-values and missing data

fEven when using Sen and Churchill's imputation [17] for missing genotypes, the effect of missing data is apparent in the permuted distribution of locus-specific P-values derived from simple reshuffling permutations such as SimplePerm.py, described above. There is a correlation (r = 0.52) between number of fraction of missing genotypes for both the classic C57BL/6J (B6) x DBA/2J (D2) RI (BXD RI) strains from The Jackson Laboratory and the new BXD RI strains [18]. Dropping imputation for missing genotypes results in an even higher (r = 0.73) for correlation with the fraction of missing data. Where genotypes themselves are being permuted and reconstructed, for instance with AIL data, there is no significant correlation between permuted locus-specific LOD score distribution and missing data in the original data set.

### Composite significance and intervals of *Hipp1a* and *Hipp9a*

**Figure 2:**
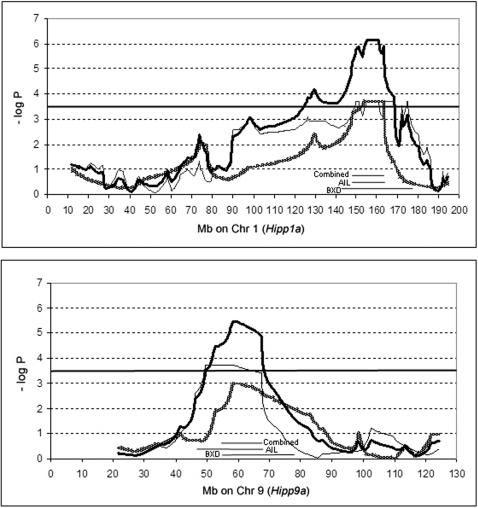
Combined mapping for *Hipp1a* and *Hipp9a.* This figure shows mapping data for the hippocampus weight loci *Hipp1a* and *Hipp9a* using 34 BXD strains (BXD; shaded line) and 679 advanced intercross animals (AIL, thin solid line) as well as the composite map using the described method (thick solid line). The genome-wide adjusted composite P = 0.05 threshold is −log P = 3.5 (dark solid horizontal line). Since 5000 permutations were used for each data set, the maximum −log P<3.7 (graphed as −log P = 3.7 for convenience) for each individual data set, so increasing the number of permutations might increase the peak combined value and slightly improve the range of the combined interval. Bars underneath the peaks are labeled AIL, BXD, and combined to indicate the l-LOD support interval of these mapping populations.

As an example of the composite mapping method, we mapped bilateral hippocampus weight in two populations, each of which has already been individually reported [14], [19]. *Hipp1a* was originally reported as significant in the BXD strain set [19]. As the re-mapping of hippocampus weight QTLs in Fig. 2 shows, we see a significant QTL on Chr. 1, but we also see a non-significant indication of a QTL on Chr. 9. (best locus-wise P = 0.001). This second QTL is individually significant in an AI population [14]. (Note that, for each population the best possible P-value is P<0.0002 since 5000 permutations were done. It may be possible to further improve composite resolution using much larger permutations.) In the case of *Hipp1a* the 1-LOD support interval (taken here as rough measure of confidence interval) is 34 Mb in the BXD strains, 17.5 Mb for the AIL, and the composite map is slightly better at 16.5 Mb, an average 51% improvement over the individual maps. For *Hipp9a* the interval was 24.5 Mb for the BXDs, 21.5 Mb for the AILs, and only 14.5 Mb for the composite map–an average improvement of 37% over the individual mapping efforts.

## Discussion

We have developed and implemented a simple method for combining QTL mapping results from multiple QTL experiments. The ready availability of dense marker maps and physical positions as well as genetic positions for markers facilitates incorporation of positional information. This method can therefore be used not only to report combined probability of the existence of a QTL locus more accurately than simply combining the best P-values for multiple mapping approaches [6]. It is also a valuable fine mapping technique and can be used in conjunction with almost any high-resolution mapping method [1]–[5] as well as for combination of multiple low-resolution mapping efforts.

### Narrowing intervals by combining mapping populations

We have demonstrated that notable improvements in genome-wide significance and QTL confidence interval are possible using our method by using the example of two hippocampus weight loci. With only two mapping populations for this phenotype, we saw improvements of 37% to 51%, in 1-LOD confidence intervals. The extensible nature of the method means that any number of available mapping populations can be added to further improve the results.

In addition, combining loci that are barely significant or narrowly miss the criteria for significance in multiple populations can clearly lead to strong observations of new loci or improved confidence in previously known loci. For instance, combining mapping results at a locus significant at a point-wise P = 0.05 for seven mapping populations or P = 0.01 for three mapping populations results in a P_combined_ = 0.0001, which is very likely significant genome-wide. Results from small mapping populations generated using the same parental strains can therefore profitably be combined using this method. Combining suggestive results (P = 0.001) with small additional results (P = 0.01) also generates much improved composite results. (P_combined_ = 0.00012)

### Crosses in multiple strains and phenotypes

While we have concentrated on multiple crosses using the same progenitor strains, it would be simple to extend this method to include crosses in other strains or, for that matter, other phenotypes measured in independent crosses. The implicit assumption in these situations is that measurement of an equivalent trait is occurring despite the cross or phenotype difference. In the case of multiple strains, this means the allele difference causing the QTL occurs in the same gene between the strains crossed in each population. Populations such as the BXD, BXA/AXB, CXB, and BXH RI strain sets, when the common allele (B6) may differ from the other strains, could fall in this category. In the case of multiple phenotypes (which could also be in multiple strains) the assumption is that the phenotypes are measuring the effect of the same polymorphism between progenitor strains for the cross.

### The importance of physical position

Genetic positions are necessary for generating QTL maps using methods such as interval mapping [20], [21]. Ultimately, however, the desired result of a QTL map is assignment of a probability that a given segment of DNA, a physical position, is involved in a phenotype. Additionally, genetic positions vary between crosses, while physical positions are constant—a desirable property when comparing between crosses is the fundamental goal.

Assigning a P-value to a physical position requires interpolation of physical positions for markers (and intermediate markers generated in interval mapping) with unknown physical positions. It also requires interpolation between these markers and the regular, set physical positions that are ultimately reported. For dense marker maps such as those typical of RI strains, the assumption that intermediate physical position can be linearly interpolated from genetic positions and flanking physical positions is reasonable. Some caution may be appropriate, however, when interpolating physical positions for widely spaced markers.

### Incorporating non-locus-specific P-values

Since locus-specific P-values are not required in the case of sufficiently large RI sets, intercrosses (F2), and backcrosses (N2), the process of explicitly computing QTL maps for each data set and performing the permutation described could be approximated by deriving P-values for each position in some more generic manner, for example from the LOD score, and combining these values as described to generate composite QTL maps and genome-wide thresholds. This is particularly useful when a simple cross with highly significant loci is being considered since the software used to sort P-values (OutParser.py) runs in approximately N^2^ time with respect to number of permutations. When converting LODs to P-values, however, under the null hypothesis of no linkage, approximately half the genome will be associated with LOD = 0 (because LODs are constrained to be positive in typical QTL maps) which should equate to P≅0.72 [8].

### Approximation of genome-wide P-value thresholds

When the distribution of permuted P-values across the genome is uniform, combination of multiple P-values for each locus should result in a uniform combined P-value distribution across the genome. We verified this empirically using a simple simulation, sampling and combining multiple observations from uniform distributions over 1000 permutations. Since in the ideal case each combined permuted QTL map will have a P-value distribution equivalent to a simple permuted QTL map, existing analytical [22] methods for estimating genome-wide P-value thresholds may be applicable where genetic lengths of the data sets being combined are similar. In most such cases, the distribution of permuted P-values is fairly uniform, so analytically determined cutoffs should provide reasonably good estimates. When a genome-wide permuted P-value is not required, the final step of combining P-values takes only a few seconds.

### Multiple mapping results from a single population

While any number of independent results can be combined using our method, it is problematic to include mapping results from the same reference population even when phenotyping was entirely independent. For instance, directly combining more than one QTL map from the same RI or congenic population is problematic. Instead it might be practical to combine the data gathered on the population as appropriate and generate a single QTL map from the combined data. This map could then be combined with any other independent mapping experiments using our method.

### Incorporation of congenic data

This method can be extended to incorporate data from a variety of data sources, including congenic and consomic data. For a polygenic trait, a test of whether a modifying locus from the introgressed parent is present in a given congenic segment addresses only the association of that region and does not contain information about the rest of the genome.

One simple approximate solution is to assume that the LOD = 0 for the remainder of the genome and apply Province's method (2001) to convert to P ≅ 0.72. This is a highly conservative assumption as our actual expectation is that there will be linkage in other parts of the genome as well for a complex trait. Fortunately, Fisher's combination test does not penalize too heavily for failure to observe association. P values for permutations could also be simulated by assigning a P-value, evenly distributed between 0 and 1, to each point outside the congenic interval. In situations where multiple congenics exist, combining the congenic interval results as a single data source might also be a reasonable approach.

### The importance of raw data archives

If meta-analysis of QTLs is limited to simple combination of maximum significance values for a QTL interval, the common practice of reporting QTL significance in terms of this maximum and of providing a figure representing the QTL interval is sufficient. For position specific analyses with joint determination of genome-wide significance thresholds, however, it is important to have access to the original data as well as the specific parameters used to perform the data analysis. Unfortunately, particularly in the older QTL literature, public access to the primary data was seldom a requirement of publication. The authors would strongly advocate that journals require public access to such data both to facilitate transparency and reproduction of results and to facilitate more sophisticated meta-analyses. All data used in generating the results for this paper is available at http://www.nervenet.org/papers/CombineQTL.html or archived at www.genenetwork.org.

### Summary

This method is a simple, easily extensible means of combining QTL mapping results from a wide variety of mapping efforts, and complements other fine mapping methods. The major advantages of this method are its simplicity, incorporation of physical position data and locus-specific P-values, and empirically estimated genome-wide P-value thresholds.

## Methods

### Assigning P-values associated with physical positions

The first step in generating a composite QTL map is to assign P-values to regular physical positions on the genome. This is done using permuted QTL maps processed to determine locus-specific P-values as described below. The details of the permutation required to generate the permuted maps are assumed to have been worked out before data is combined.

We use locus-specific P-values because in certain data sources such as advanced intercrosses and small RI sets, the correspondence between the likelihood typically reported in mapping output (a LOD score for R/qtl, [23] for instance, or a LRS score in the case of WebQTL [24], [25]) and the P-value, as determined by generation of permuted QTL maps, is not constant with respect to position. This can be related to missing data patterns, distribution of alleles and non-syntenic association.

Once all of the permuted maps are generated, we assign locus-specific p-values to regularly spaced physical positions on each chromosome for the original data. In order to derive locus-specific P-values from raw LOD scores we let be the observed LOD at locus j. For each permutation, L_perm_(i,j) is the observed LOD at locus j in permutation replicate i. The locus-specific P-value for locus j is P(j) = proportion{ L_perm_(i,j)∃L_obs_(j)}. This is the proportion of the permutation replicates that have a LOD score greater than or equal to the observed LOD score at locus j.

P-values at regular intervals were based on the known physical and genetic positions of markers and their P-values as computed above. First we linearly interpolated missing physical positions using flanking genetic positions for the nearest markers with complete information (assuming 0 cM and 0 Mb for the proximal end of the chromosome and assuming linear extension for the distal end of the chromosome using the ratio of physical to genetic distance of the nearest proximal markers.) After we obtained a complete set of physical positions, we linearly interpolated P values at regular intervals on a grid of physical positions, allowing values from the final marker to the end of the chromosome to equal those at the final marker.

For sparsely genotyped data sets it is more accurate to linearly interpolate using LOD scores based on an interval map and derive locus-specific P-values from the interpolated LOD scores, though this method is slower than the one described above. As genotype density increases the order of these steps has less effect. We tested the effect of this difference for our BXD RI data at 300 markers and the average percent difference in P-value between methods was only 12%. For all model data sets except our F2 data set, which is sparsely genotyped, we therefore calculated locus-specific P-values first. We provide an implementation for both methods.

### Sample data sets and phenotypes

For calculating the LOD score equivalent to a locus-specific P = 0.05 (referred to as 95% LOD) in Fig. 1, we used raw body weight measurements from a previously described intercross [19], as well as other previously described data [19] from the classic BXD recombinant inbred (RI) strains [26], [27], which are formed by repeated intercrossing and inbreeding the progeny of C57BL/6J and DBA/2J. In addition, we gathered new body weight data for a recently described [18] set of BXD RI strains and from an advanced intercross [14] using similar criteria.

For composite mapping of hippocampus weight QTLs shown in Fig. 2, we used previously described observations in the classic BXD RI strains [19] and observations made using the same protocol from an advanced intercross, the construction of which is described elsewhere [14]. For this example of combined mapping data, we used the method and software described below.

### Combining QTL data sources

At the end of the process outlined above, each data source will be represented by a set of locus-specific P-values associated with regular and defined physical positions. For each of these positions, j, there will be k associated P-values, one from each data source. Since by Fisher's combination test −2 Σ ln P(k,j) for all k data sources at locus j follows a chi-squared distribution with 2k degrees of freedom in the case of the null hypothesis, (no association between phenotype and genotype) we can easily associate each position with a composite locus-wise P-value, P_combined_(j).

### Generating genome-wide P-value thresholds

The combined map gives us a locus-specific combined P-value for any given locus, P_combined_(j), but it is also important to have a genome-wide threshold for error control at a specified genome-wide P-value. Genome-wide LOD score thresholds for QTL experiments are typically generated by permuting genotype or phenotype, re-running the mapping phase of the experiment for a large number of permutations [28], and creating an ordered list of the highest LOD score in each permutation. The genome-wide adjusted P-value is the proportion of this ordered list that is greater than or equal to a given LOD score in the original QTL map.

The approach here is similar except that we are first converting LOD scores to P-values for each permutation i. This is done as described above except that observed LOD score at locus j, is the value of the LOD score for a particular permutation in i, designated i_fixed,_ for which locus-specific P-values are currently being calculated. We will term the LOD score for i_fixed _at locus j L(i_fixed_,j) and will designate the permuted LOD scores for all permutations except i_fixed_ as L_perm_(i',j). This process is an excellent approximation of the locus-specific P-value that would result from generating a set of permutations of each i_fixed_, assuming that L_perm_ (i',j) has a distribution equivalent to L_perm_(i,j), which should be true for large i.

In other words, for each permutation in i, the locus-specific P-value at j is
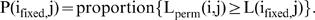
After calculating locus specific P-values for all i permutations at all j positions, P(i,j), in all k data sets, we can estimate the genome-wide adjusted P-value. We do this by sampling a permuted map of locus specific P-values, P(i), for each of k data sets and calculating the combined P-value, P_perm, combined_(j), for each corresponding locus between sampled permuted maps as described above using Fisher's combination test.

We then record the minimum P_perm, ombined_(j) value from each combined map. This permutation is repeated to create an ordered list of best P-values, P_best_. For a given P_combined_(j) value in the original data set, the genome-wide adjusted P-value, P_adjusted_, for the combined data is
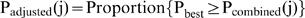
Typically, however, the ordered list is simply used to calculate a genome-wide threshold, often set the 95^th^ percentile value for P_best_ (genome-wide adjusted P = 0.05).

### Software

All software described is available at http://www.nervenet.org/papers/CombineQTL.html. For the customized analyses above, we wrote scripts using Python 2.4 as well as simple R scripts to automate use of R/qtl. Further integration with R/qtl as well as other QTL mapping programs should be relatively straightforward, providing that automation of the mapping functionality itself is achievable as it is with R/qtl. Permutations generated for advanced intercross lines were created using a custom python script designed for the purpose, the operation of which is described elsewhere [14]. Permutation of populations with independent samples, such as F2, N2, and RI (phenotypic averages) populations can all be accomplished by simply shuffling the list of phenotypes. A Python script that does this and writes a file of permuted phenotypes, **SimplePerm.py**, is included.

We wrote a set of two Python scripts, **OutParser.py**, **and OutParserLI.py** to parse the mapping output files from R/qtl and to generate a map of locus-specific P-values at regular, user defined intervals on a physical scale. These versions differ only in that OutParser.py calculates locus-specific P-values at markers then interpolates to regular positions, while OutParserLI.py (**L**OD **I**nterval) reverses the order of these steps. OutParserLI.py is somewhat slower but preferable for sparsely genotyped data sets. These scripts require a data file, **markerphyspos.txt** in the example files, which contains genetic positions for each marker (and/or interpolated marker if interval mapping has been performed) in the R/qtl output file as well as physical positions for as many markers as possible. Because the locus-specific P-values will often be used in combination with a similar set of values, defined at the same intervals, in another population (see description of **CombineMaps.py** below), the P values at the ends of each chromosome must be handled carefully. (If different data sources are associated with different markers, the beginning and ending points of each chromosome may well be different.) The assumption we have made is to repeat the most proximal actual marker value to the most proximal end of a given chromosome and likewise to repeat the most distal value to the distal end. Further, each chromosome is defined as 200 Mb in length (This is just slightly longer than the longest actual mouse chromosome. The user can adjust this value.) to ensure that values from each new data set can be easily accommodated and to eliminate the need for a separate file specifying length of each chromosome. This is also a convenient convention for plotting composite maps as the “unused” regions can be easily masked and graphs from multiple data sources, which may differ slightly in chromosome length and markers, can easily be lined up and overlaid.

**CombineMaps.py** combines QTL maps of locus-specific P-values from different data sources. In order to generate combined maps of original data the program simply applies Fisher's combination test to the locus-specific P-values at equivalent physical positions for each defined position in the genome. The program also implements the method described above for finding genome-wide adjusted P-values. It does this by sampling with replacement from the set of permuted QTL maps for each data source. These maps are combined as described above and the best combined locus-specific P-value is recorded. This list of best P-values can be sorted (easily done in Excel or in Unix using “sort –n filename>sortedfilename”) to allow genome-wide interpretation of locus-specific P-values.
